# Phase I study to assess the effect of adavosertib (AZD1775) on the pharmacokinetics of substrates of CYP1A2, CYP2C19, and CYP3A in patients with advanced solid tumors

**DOI:** 10.1007/s00280-023-04554-3

**Published:** 2023-07-02

**Authors:** Mats Någård, Mei-Lin Ah-See, James Strauss, Trisha Wise-Draper, Howard P. Safran, Laura Nadeau, William J. Edenfield, Lionel D. Lewis, Lone H. Ottesen, Yan Li, Ganesh M. Mugundu

**Affiliations:** 1 Clinical Pharmacology and Quantitative Pharmacology, Clinical Pharmacology and Safety Sciences, AstraZeneca, One MedImmune Way, Gaithersburg, MD 20878 USA; 2 Late-Stage Development, Oncology R&D, AstraZeneca, Cambridge, UK; 3 Mary Crowley Cancer Research Center, Dallas, TX USA; 4 University of Cincinnati Cancer Center, Cincinnati, OH USA; 5 Rhode Island Hospital, Lifespan Cancer Institute, Providence, RI USA; 6 Beaumont Cancer Center, Royal Oak, MI USA; 7 Institute for Translational Oncology Research, Greenville, SC USA; 8 The Dartmouth Cancer Center and Dartmouth-Hitchcock Medical Center, Lebanon, NH USA; 9 Integrated Bioanalysis, Clinical Pharmacology and Quantitative Pharmacology, BioPharmaceuticals R&D, AstraZeneca, Boston, MA USA; 10 Clinical Pharmacology and Quantitative Pharmacology, R&D Clinical Pharmacology and Safety Sciences, AstraZeneca, Waltham, MA USA

**Keywords:** Adavosertib, AZD1775, Pharmacokinetics, CYP1A2, CYP2C19, CYP3A

## Abstract

**Purpose:**

Adavosertib may alter exposure to substrates of the cytochrome P450 (CYP) family of enzymes. This study assessed its effect on the pharmacokinetics of a cocktail of probe substrates for CYP3A (midazolam), CYP2C19 (omeprazole), and CYP1A2 (caffeine).

**Methods:**

Period 1: patients with locally advanced or metastatic solid tumors received ‘cocktail’: caffeine 200 mg, omeprazole 20 mg, and midazolam 2 mg (single dose); period 2: after 7- to 14-day washout, patients received adavosertib 225 mg twice daily on days 1–3 (five doses), with cocktail on day 3. After cocktail alone or in combination with adavosertib administration, 24-h pharmacokinetic sampling occurred for probe substrates and their respective metabolites paraxanthine, 5-hydroxyomeprazole (5-HO), and 1′-hydroxymidazolam (1′-HM). Safety was assessed throughout.

**Results:**

Of 33 patients (median age 60.0 years, range 41–83) receiving cocktail, 30 received adavosertib. Adavosertib co-administration increased caffeine, omeprazole, and midazolam exposure by 49%, 80%, and 55% (AUC_0–12_), respectively; AUC_0–t_ increased by 61%, 98%, and 55%. Maximum plasma drug concentration (C_max_) increased by 4%, 46%, and 39%. Adavosertib co-administration increased 5-HO and 1′-HM exposure by 43% and 54% (AUC_0–12_) and 49% and 58% (AUC0–t), respectively; paraxanthine exposure was unchanged. Adavosertib co-administration decreased C_max_ for paraxanthine and 5–HO by 19% and 7%; C_max_ increased by 33% for 1′-HM. After receiving adavosertib, 19 (63%) patients had treatment-related adverse events (six [20%] grade ≥ 3).

**Conclusion:**

Adavosertib (225 mg bid) is a weak inhibitor of CYP1A2, CYP2C19, and CYP3A.

**ClinicalTrials.gov:**

NCT03333824

**Supplementary Information:**

The online version contains supplementary material available at 10.1007/s00280-023-04554-3.

## Introduction

Cyclin-dependent kinase 1 (CDK1; also known as cell division cycle 2 protein [CDC2]) drives a cell from the G2 phase of the cell cycle into mitosis. In response to DNA damage, the nuclear tyrosine kinase Wee1 inhibits CDK1 to prevent the cell from dividing until the damaged DNA is repaired (G2 checkpoint arrest) [1]. CDK2 drives a cell into, and through, the S phase of the cell cycle, during which the genome is duplicated in preparation for cell division; inhibition of Wee1 is expected to cause aberrantly high CDK2 activity in S-phase cells that, in turn, leads to unstable DNA replication structures and, ultimately, DNA damage [2].

Fully functional CDK1- and CDK2-mediated checkpoints are necessary for the DNA damage response (DDR) to minimize replication stress for proliferating cells [3–6]. Adavosertib (AZD1775) is a highly selective ATP-competitive small-molecule inhibitor of Wee1, with a half-maximal inhibitory concentration (IC_50_) of 5.2 nmol/L in in vitro kinase assays [7, 8]. Inhibition of Wee1 releases tumor cells from DNA-damage-induced arrest at the G2/M boundary, so that unrepaired DNA damage may be taken into mitosis (M phase); as cancer cells show higher levels of endogenous damage than normal cells, as well as exhibiting loss of one or more DDR capabilities, this is predicted to preferentially enhance cancer cell death through mitotic catastrophe compared with normal cells [2, 9].

Adavosertib has been evaluated as mono- and combination therapy with chemotherapy, olaparib, and durvalumab in numerous phase I and II studies in patients with a wide variety of solid tumors [10–15]. Preclinical (in vitro) studies indicate that adavosertib is metabolized by, and is both a substrate for and an inhibitor of, certain enzymes of the cytochrome P450 (CYP) family [16]. Metabolism of adavosertib is predominantly by CYP3A, with a flavin-containing monooxygenase (FMO) 3 and/or FMO5 component (AstraZeneca, data on file, 2022). Adavosertib is also a weak reversible inhibitor (IC_50_ 14 μM) and a time-dependent (irreversible) inhibitor of CYP3A (maximal inactivation [k_inact_] 0.061/min, concentration at 50% of k_inact_ 6.04 μM); modeling data predicted an eight- to tenfold increase in the exposure of sensitive CYP3A substrates when administered with adavosertib (250 mg twice daily [bid] for five doses; AstraZeneca, data on file, 2020) [16]. Adavosertib is a weak inducer of CYP1A2 (39% increase in activity of positive control; AstraZeneca, data on file, 2022) [16].

The primary objective of this prospective, two-period, open-label, drug–drug interaction (DDI) study (NCT03333824) was to determine whether there was a clinically significant increase of exposure to substrates for CYP1A2 (caffeine), CYP2C19 (omeprazole), and CYP3A (midazolam) in the presence of adavosertib, and if co-administration of the probe CYP substrates had a clinically meaningful effect on adavosertib pharmacokinetics (PKs) [17]. The doses of the probe drugs (midazolam: 1 mL of 2 mg/mL syrup; omeprazole: 20 mg capsules; caffeine: 200 mg tablet) included in the cocktail were previously validated in this cocktail [18].

## Methods

### Objectives

The primary study objective was to assess the effect of adavosertib on the PKs of probe substrates for CYP3A (midazolam), CYP2C19 (omeprazole), and CYP1A2 (caffeine). The secondary study objectives were to: describe the PKs of midazolam, omeprazole, and caffeine and their respective metabolites (1ʹ-hydroxymidazolam [1ʹ-HM], 5-hydroxyomeprazole [5-HO], and paraxanthine) in the absence and presence of adavosertib; describe the PKs of adavosertib; and observe the clinical and laboratory safety and tolerability of adavosertib.

### Study design

**Fig. 1:**
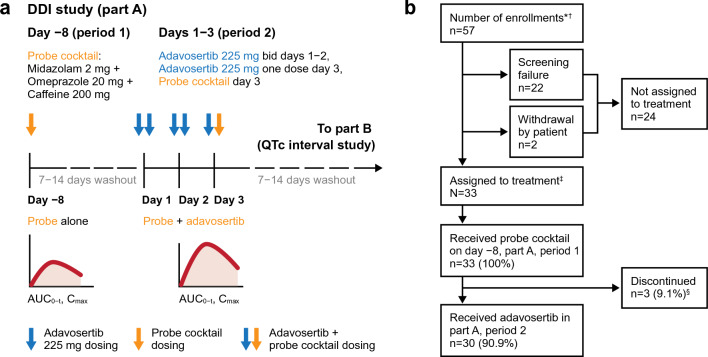
**a** Study design and **b** patient disposition flow chart. This manuscript focuses on pharmacokinetic data from part A of NCT03333824; part B of NCT03333824 is an investigation of the effect of adavosertib on the QT interval, results of which are reported separately. *Informed consent received; ^†^A number of patients enrolled more than once; there were 49 unique enrollments; ^‡^Study treatment refers to treatment with either cocktail or adavosertib; ^§^One each as a result of death (pancreatic cancer), study termination by the sponsor, and withdrawal by the patient. *bid* twice daily

This manuscript focuses on PK data (part A, periods 1 and 2) from NCT03333824, which was conducted at seven clinical sites in the USA. Part B of NCT03333824 was an investigation of the effect of adavosertib on the electrocardiogram QT interval, the results of which are reported separately. The study was of a prospective, open-label, non-randomized, sequential, two-period design (Fig. 1).

Treatment started with single-dose administration of the probe cocktail of caffeine (200 mg tablet), omeprazole (20 mg capsule), and midazolam (1 mL of 2 mg/mL syrup formulation) on day − 8, followed by PK sampling for 24 h (period 1) and a washout period of 7–14 days. In period 2, adavosertib (225 mg; 3 × 75 mg capsules) was administered bid until steady state for 2.5 days (total of five doses; maximum tolerated/recommended phase II dose for combination therapy [19]), and the final dose was administered in combination with single doses of the probe cocktail drugs on the morning of day 3, followed by PK sampling for 24 h. Venous blood samples were taken immediately pre-dose, as well as post-dose at 15, 30, and 45 min and 1, 2, 3, 4, 6, 8, 10, 12, and 24 h following a single dose of the cocktail alone on day − 8 or the cocktail with adavosertib 225 mg on day 3. Patients were screened 28 days before the first adavosertib dose and within 14–21 days before the first cocktail administration. Patients were required to fast from 2 h before until 2 h after the probe cocktail drug administration, as well as each administration of adavosertib alone or in combination with the cocktail.

Patients received granisetron 1 mg orally as anti-emetic prophylaxis 30 min prior to administration of the cocktail drugs or adavosertib capsules. Water intake was prohibited from 1 h before until 1 h after administration of the probe cocktail alone or with adavosertib (days − 8 and 3, respectively), excluding the 240 mL of water used for administration of adavosertib and the cocktail. Dexamethasone was prohibited until after the last PK sample was drawn; prochlorperazine, promethazine, and/or lorazepam could be used as required to treat nausea/vomiting.

The study was performed in accordance with the ethical principles of the Declaration of Helsinki and is consistent with the International Council for Harmonisation’s Good Clinical Practice, applicable regulatory requirements, and the AstraZeneca policy on bioethics [20].

### Patients

Patients were eligible for the study if they met the following inclusion criteria: presence of a histologically or cytologically confirmed locally advanced or metastatic solid tumor, excluding lymphoma, for which standard therapy did not exist or had proven ineffective or intolerable; any prior palliative radiation must have been completed at least 7 days prior to the start of study treatment, and patients must have recovered from any acute adverse effects prior to the start of study treatment; Eastern Cooperative Oncology Group performance status of 0 or 1; no abnormalities in laboratory values within 7 days of initiation of study treatment; ≥ 18 years of age. Key exclusion criteria included: patients who were suffering from conditions that were likely to adversely affect gastrointestinal motility and/or transit or drug absorption; patients taking prescription medicines or foods known to interfere with the PKs of adavosertib or the probe cocktail drugs (see Supplementary Methods for restricted medications and additional exclusion criteria).

### Pharmacokinetic assessment

After cocktail alone (day − 8) and with adavosertib co-administration (period 2 day 3), 24-h venous blood sampling took place for PK measurements of caffeine, omeprazole, midazolam*,* and their respective metabolites paraxanthine, 5-HO, and 1ʹ-HM. This sampling period was selected based on the half-lives of the probe substrates. The PK parameters measured included maximum plasma drug concentration (C_max_), area under the plasma concentration–time curve (AUC) from time zero to infinity (AUC_0–∞_), AUC from time zero to time of the last quantifiable concentration (AUC_0–t_), time to reach maximum plasma concentration (t_max_), plasma terminal half-life (t_½_), elimination rate constant (λ_z_), apparent clearance (CL/F), and apparent volume of distribution (V_z_/F) for the cocktail parent compounds (midazolam, omeprazole, and caffeine).

AUC from time zero to 12 h (AUC_0–12_), AUC_0–t_, t_max_, C_max_, t_½_, and λ_z_ were determined for the cocktail metabolites (1ʹ-HM, 5–HO, and paraxanthine), and AUC and C_max_ ratios were determined in relation to the parent compounds. Following adavosertib administration on period 2 day 3, PK parameters for adavosertib measured included AUC_0–12_, t_max_, C_max_, minimum plasma drug concentration (C_min_), apparent average plasma concentration over a dosing interval (C_avg_), apparent clearance at steady state (CL_ss_/F), and fluctuation index over a dosing interval (FI).

Plasma concentrations for caffeine, paraxanthine, omeprazole, 5-HO, midazolam, and 1ʹ­HM were all determined by high-performance liquid chromatography–tandem mass spectrometry (HPLC–MS/MS) at Labcorp Bioanalytical Laboratories (Madison, WI, USA).

Plasma concentrations of caffeine and paraxanthine collected with K_2_EDTA as anticoagulant were determined by HPLC–MS/MS detection simultaneously. The standard curves of caffeine and paraxanthine both ranged from 25.0 to 20,000 ng/mL. Precision (percentage coefficient of variance [%CV]) and accuracy (percentage bias) for the quality control (QC) samples were ≤ 7.0% and ≤ 7.5% CV and within − 3.1% to − 1.8% and − 4.9% to − 1.8% bias for caffeine and paraxanthine, respectively. The lower limit of quantification (LLOQ) was 25.0 ng/mL for caffeine and paraxanthine.

Plasma concentrations of omeprazole and 5-HO were collected with lithium heparin as anticoagulant and were determined by HPLC–MS/MS detection simultaneously. The standard curves of omeprazole and 5-HO both ranged from 20.0 to 20,000 nmol/L. Precision (%CV) and accuracy (percentage bias) for the QC samples were ≤ 3.9% and ≤ 4.4% CV and within 0.0 − 9.0% and − 1.9% to 2.2% bias for omeprazole and 5-HO, respectively. The LLOQ was 20.0 nM for omeprazole and 5-HO.

Plasma concentrations of midazolam and 1ʹ-HM were collected with K_2_EDTA as anticoagulant and were determined by HPLC–MS/MS detection simultaneously. The standard curves of midazolam and 1ʹ-HM both ranged from 0.100 to 100 ng/mL. Precision (%CV) and accuracy (percentage bias) for the QC samples were ≤ 6.4% and ≤ 5.1% CV and within − 0.8% to 1.0% and − 1.0% to 0.0% bias for midazolam and 1ʹ-HM, respectively. The LLOQ was 0.1 ng/mL for midazolam and 1ʹ-HM.

The concentration of adavosertib in human plasma was determined by Labcorp Bioanalytical Laboratories using the same validated method described previously [21]. The assay had a linear dynamic range of 2–1000 ng/mL, with an LLOQ of 2 ng/mL [22].

All plasma samples were stored at − 70 °C (or below) prior to analysis and analyzed within the validated time frame.

### Safety and tolerability assessments

Safety was appraised throughout the study by the assessment of clinical and laboratory adverse events (AEs; graded by Common Terminology Criteria for Adverse Events [CTCAE], version 4.03), physical examination, and evaluation of vital signs and laboratory data (clinical chemistry and hematology).

### Statistical analyses

No formal sample size estimation was conducted. Based on an estimate of within-patient standard deviation of 0.294 and assuming a true interaction effect of 100%, it was estimated that 20 evaluable patients would provide 80% power to show that the 90% confidence interval (CI) for the adavosertib effect lies entirely below 2.67, i.e. it would rule out a 167% increase of exposure in the presence of adavosertib. The number of patients was based on careful clinical consideration to gain adequate information on the primary endpoints while exposing as few patients as possible to study procedures. Enrollment of approximately 30 patients, with a target of 20 evaluable patients, was considered adequate and sufficient to meet the objectives of this study.

The safety analysis set included all patients who received at least one dose of study treatment (adavosertib or probe cocktail drugs). Patients were evaluated according to the treatment received. A treatment-emergent AE was defined as an AE with its start date and time on or after the first dose of probe cocktail on day − 8 up to and including 30 days after the last dose date of adavosertib, or, for pre-existing pre-treatment AEs, the date on which they worsened in severity after the first dose of study treatment. The PK analysis set included all dosed patients who had at least one quantifiable plasma concentration for any of the cocktail drugs (or metabolites) or adavosertib collected post-dose without protocol deviations or events that could affect the PK analysis.

PK parameters were derived using non-compartmental methods with Phoenix^®^ WinNonlin^®^ version 6.4 (Certara, LP, Princeton, NJ, USA). All descriptive and inferential statistical computations were performed using SAS^®^ version 9.1 (SAS Institute, Cary, NC, USA). Estimates of the mean difference between treatments (adavosertib + probe cocktail substrate compared with probe cocktail substrate alone) and corresponding 90% CIs were calculated using a linear mixed-effects model, with a fixed effect for treatment and a random effect for patient. The natural-log-transformed PK parameters (C_max_, AUC_0-∞_, and AUC_0–t_) of the cocktail parent compounds and metabolites were used in the mixed-effects models as dependent variables. The mean differences and CIs were back transformed to the original scale to give estimates of the ratios and the associated 90% CIs; additionally, back-transformed geometric means, together with 95% CIs, were estimated and are presented by treatment.

Results for AUC_0–12_ and C_max_ obtained following adavosertib administration on period 2 day 3 were compared with those obtained on period 2 day 3 following the same adavosertib dosage schedule but without cocktail (AstraZeneca, data on file, 2022) to probe the potential effects of the cocktail drugs and to obtain an estimate of intra-patient adavosertib variability.

## Results

### Patients

Of 57 patients enrolled (from 49 unique patients, as several patients enrolled more than once because of rescreening), 33 were assigned to treatment (see Fig. 1 for patient disposition flow chart). Patient baseline characteristics are shown in Table 1; the median age of patients assigned to treatment was 60 years (range 41–83), and the gender distribution was balanced. Six patients used disallowed concomitant medications, including three patients who were taking a weak CYP3A inhibitor that could have affected the PK results for midazolam and its metabolite 1ʹ-HM (two patients took amlodipine, one patient took alprazolam); these patients had their midazolam and 1ʹ-HM PK results excluded. Details of the number of patients with evaluable data for each drug/period are provided in Supplementary Table S1.

**Table 1:** Patient demographics and disease characteristics (safety analysis set)

Characteristic	Safety analysis set (*N* = 33)
Age, years	
Mean (SD)	60.3 (8.8)
Median (range)	60 (41–83)
Age group, *n* (%)	
40– < 50 years	5 (15.2)
50– < 65 years	20 (60.6)
≥ 65 years	8 (24.2)
Sex, *n* (%)	
Male	15 (45.5)
Female	18 (54.5)
Race, *n* (%)	
Asian	1 (3.0)
Black or African American	4 (12.1)
White	28 (84.8)
Body mass index, kg/m^2^	
*n*	29
Mean (SD)	25.8 (4.9)
Median (range)	25.0 (17.5–36.9)
ECOG performance status, *n* (%)	
(0) Normal activity	15 (45.5)
(1) Restricted activity	18 (54.5)
Primary tumor location, *n* (%)^a^	
Pancreas	5 (15.2)
Colon	4 (12.1)
Breast	3 (9.1)
Lung	3 (9.1)
Ovary	3 (9.1)
Peritoneum	3 (9.1)
Other^b^	12 (36.4)

*ECOG* performance status and overall disease classification are based on assessments at baseline. Primary tumor location and histology type are based on assessments at primary diagnosis. ^a^All patients enrolled in this study had disease progression to unresectable recurrent/metastatic cancers since diagnosis. ^b^Other includes appendix, cervix uteri, gastric cardia, head and neck, head and neck – oral cavity, larynx, pancreatic head, prostate gland, rectum, small intestine, submandibular gland, and uterus (all *n* = 1). *ECOG* Eastern Cooperative Oncology Group, *SD* standard deviation

### Pharmacokinetic assessments

Pharmacokinetic parameter estimates for substrates of CYP1A2 (caffeine), CYP2C19 (omeprazole), and CYP3A (midazolam) and their metabolites (paraxanthine, 5-HO, and 1ʹ-HM, respectively) in the presence and absence of adavosertib are shown in Table 2.

**Table 2:** Pharmacokinetic parameter estimates for substrates of CYP1A2 (caffeine), CYP2C19 (omeprazole), and CYP3A (midazolam) and their metabolites (paraxanthine, 5-HO, and 1ʹ-HM, respectively) in the presence and absence of adavosertib 225 mg bid (pharmacokinetic analysis set)

	Point estimates of the geometric LS mean ratios S + AD/S, % (90% CI)			Median t_max_, h (range)		Mean t_½_ h (SD)		Mean CL/F, L/h (SD)		Mean V_z_/F, L (SD)		Mean MR					
												AUC		AUC_0–t_		C_max_	
	AUC, ng·h/mL	AUC_0–t_, ng·h/mL	C_max_, ng/mL	− AD	+ AD	− AD	+ AD	− AD	+ AD	− AD	+ AD	− AD	+ AD	− AD	+ AD	− AD	+ AD
Caffeine^a^	149.1 (131.3–169.3)	160.8 (143.3–180.5)	103.8 (92.2–116.9)	0.5 (0.3–3.0)	0.7 (0.3–3.0)	6.4 (3.8)	13.5 (6.8)	5.7 (2.5)	3.8 (1.9)	39.3 (15.0)	37.9 (12.1)	NA					
Omeprazole	180.2 (145.7–222.9)	198.1 (160.3–244.7)	145.5 (114.0–185.7)	3.0 (0.7–6.0)	3.9 (0.5–8.0)	2.0 (1.0)	2.5 (1.3)	13.3 (10.5)	7.6 (7.3)	28.3 (16.6)	19.8 (10.1)	NA					
Midazolam	155.3 (138.6–173.9)	155.2 (140.5–171.5)	138.5 (118.9–161.3)	0.5 (0.2–1.0)	0.5 (0.2–1.0)	5.5 (2.6)	6.6 (3.0)	59.0 (34.3)	39.7 (14.5)	409.9 (211.5)	364.1 (182.7)	NA					
Paraxanthine^b^	NC	NC	81.2 (74.6–88.5)	8.0 (4.0–11.8)	9.9 (6.0–24.9)	7.0 (2.7)	12.2 (5.4)	NA				0.65	NC	0.54	0.32	0.23	0.16
5-HO	143.0 (123.8–165.0)	148.8 (129.6–170.8)	92.9 (78.6–109.8)	3.0 (0.7–6.0)	4.0 (1.8–7.8)	2.7 (1.7)	4.5 (4.7)	NA				0.50	0.46	0.73	0.50	0.53	0.30
1ʹ-HM	153.5 (131.0–179.8)	158.2 (139.1–180.0)	133.4 (106.5–167.1)	0.5 (0.3–2.1)	0.5 (0.2–1.0)	7.2 (6.1)	7.6 (7.1)	NA				0.41	0.45	0.43	0.44	0.47	0.43

Patient numbers for cocktail without AD (period 1) were: caffeine (AUC: 22; AUC_0–t_, C_max_, t_max_, and t_½_: 25), omeprazole (AUC and t_½_: 21; AUC_0–t_, C_max_, and t_max_: 27), midazolam (AUC and t_½_: 22; AUC_0–t_, C_max_, and t_max_: 23), paraxanthine (AUC: 10; t_½_: 14; AUC_0–t_, C_max_, and t_max_: 19), 5-HO (AUC: 22; t_½_: 24; AUC_0–t_, C_max_, and t_max_: 28), and 1'-HM (AUC: 20; t_½_: 22; AUC0–t, C_max_, and t_max_: 24). Patient numbers for cocktail with AD (period 2) were: caffeine (AUC: 8; AUC_0–t_ and t_½_: 18; C_max_ and t_max_: 19), omeprazole (AUC and t_½_: 15; AUC_0–t_: 18; C_max_ and t_max_: 20), midazolam (AUC and t_½_: 18; AUC_0–t_, C_max_, and t_max_: 19), paraxanthine (AUC: 1; t_½_: 5; AUC_0–t_: 15; C_max_ and t_max_: 16), 5-HO (AUC: 13; t_½_: 17; AUC_0–t_: 18; C_max_ and t_max_: 20), and 1'-HM (AUC: 17; t_½_: 18; AUC_0–t_, C_max_ and t_max_: 19). ^a^Caffeine AUC was only reliably characterized in eight patients receiving cocktail with AD in period 2. Caffeine AUC increased in all seven patients with paired data for both periods; results were considered representative of the data. ^b^Paraxanthine AUC was only reliably characterized in 10 patients in period 1 (cocktail only) and one patient in period 2 (cocktail + AD); thus, the treatment comparison for AUC was not considered scientifically meaningful. *1ʹ-HM* 1ʹ-hydroxymidazolam, *5-HO* 5-hydroxyomeprazole, *AD* adavosertib, *AUC* area under the plasma concentration–time curve, *AUC*_*0–t*_ area under the plasma concentration–time curve from time zero to time of the last quantifiable concentration, *CI* confidence interval, *CL/F* apparent clearance, *C*_*max*_ maximum plasma drug concentration, *CYP* cytochrome P450, *LS* least-squares, *MR* metabolic ratio in relation to parent compound, *NA* not available, *NC* not calculable, *S* + *AD/S* substrate (or compound) + adavosertib compared with substrate or compound alone, *SD* standard deviation, *t*_*½*_ plasma terminal half-life, *t*_*max*_ time to reach maximum plasma concentration, *V*_*z*_*/F* apparent volume of distribution

Co-administration of adavosertib increased caffeine total exposure by 49% (AUC_0–12_) to 61% (AUC_0–t_; Table 2). Caffeine AUC was only reliably characterized in eight patients in period 2. With the exception of one patient, caffeine AUC_0–12_ increased following co-administration of adavosertib in all patients with paired data for both periods; hence, the results obtained for AUC_0–12_ from this inferential analysis were considered to be representative of the data.

Paraxanthine AUC_0–12_ was only reliably characterized in 10 patients in period 1 and one patient in period 2. Thus, the treatment comparison for AUC_0–12_ was not considered meaningful.

Co-administration of adavosertib increased omeprazole total exposure by 80% (AUC_0–12_) to 98% (AUC_0–t_) and increased C_max_ by 46% (Table 2).

Co-administration of adavosertib increased midazolam total exposure by 55% (AUC_0–12_ and AUC_0–t_; Table 2).

**Fig. 2:**
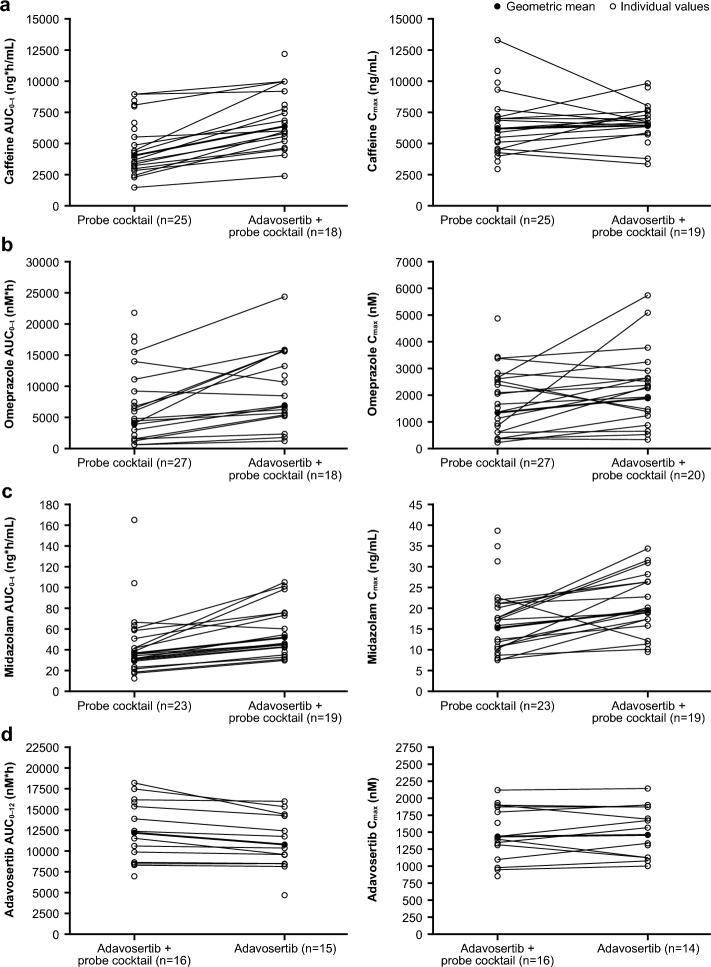
Individual and geometric mean AUC_0–t_ and C_max_ of **a** caffeine, **b** omeprazole, and **c** midazolam alone versus each cocktail drug plus adavosertib, and **d** individual and geometric mean of adavosertib plus cocktail (part A) versus adavosertib alone (part B). *Individual patient and geometric mean AUC_0–t_ and C_max_ in the presence and absence of adavosertib 225 mg bid for caffeine (*n* = 25), omeprazole (*n* = 27), and midazolam (*n* = 23); ^†^Adavosertib + cocktail: caffeine (200 mg tablet), omeprazole (20 mg capsule), and midazolam (1 mL of 2 mg/mL syrup formulation) on day 3 and adavosertib 225 mg (3 × 75 mg capsules) on day 3 (part A). Adavosertib: adavosertib 225 mg (3 × 75 mg capsules) bid on days 1 and 2 and once on day 3 (part B/pharmacodynamic study). *AUC*_*0–12*_ area under the plasma concentration–time curve from time zero to 12 h, *AUC*_*0–t*_ area under the plasma concentration–time curve from time zero to time of the last quantifiable concentration, *C*_*max*_ maximum plasma drug concentration

The individual and geometric mean AUC_0–12_ and C_max_ of adavosertib plus cocktail (part A) versus adavosertib alone (part B), and the individual and geometric mean AUC_0–t_ and C_max_ of caffeine, omeprazole, and midazolam alone versus each cocktail drug plus adavosertib, are shown in Fig. 2.

**Fig. 3:**
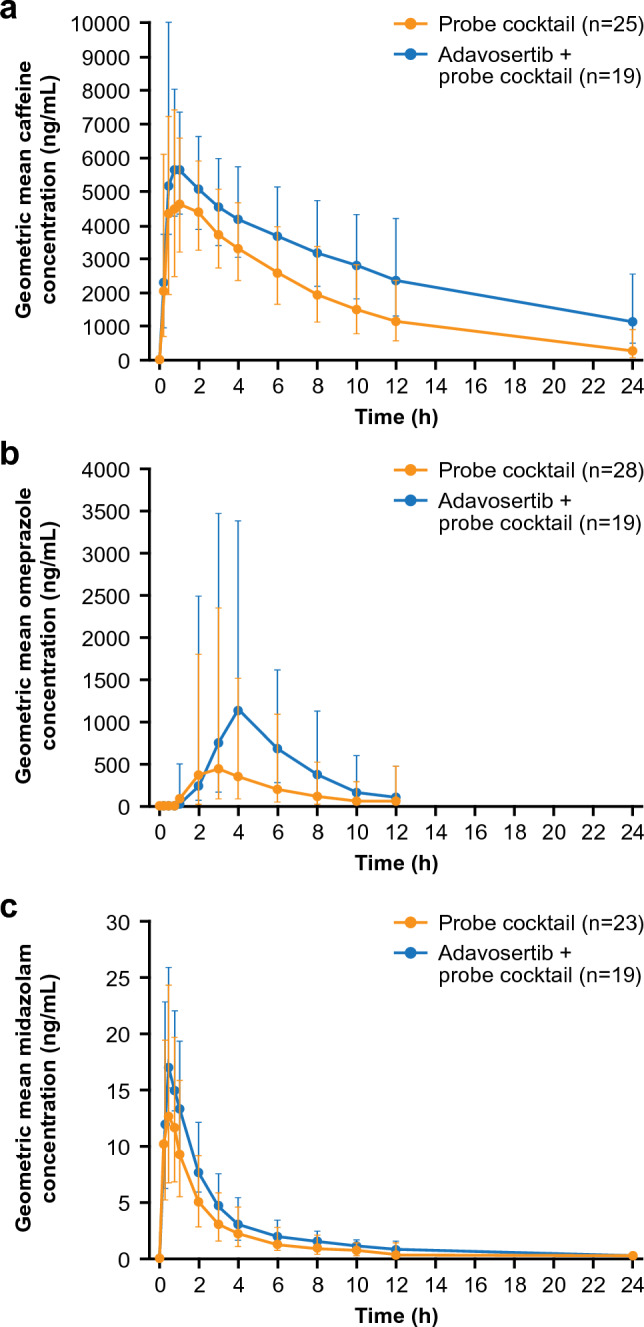
Geometric mean (± SD) plasma concentration of **a** caffeine, **b** omeprazole, and **c** midazolam ± adavosertib versus time. Exponential of (mean of log concentration ± SD of log concentration). Probe cocktail: caffeine (200 mg tablet), omeprazole (20 mg capsule), and midazolam (1 mL of 2 mg/mL syrup formulation) on day − 8. Adavosertib + cocktail: cocktail and adavosertib 225 mg (3 × 75 mg capsules) on day 3. *SD* standard deviation

The geometric mean plasma concentrations of caffeine, omeprazole, and midazolam versus time, with and without adavosertib, are shown in Fig. 3. In the presence of adavosertib, the rate of paraxanthine formation appeared to be decreased, resulting in lower mean concentrations and a lower rate of elimination over the sampling period (Supplementary Figure S1).

The PK parameters of adavosertib following co-administration of cocktail are shown in Table 3. Point estimates of the AUC_0–12_ and C_max_ geometric least-squares (LS) mean ratios were 108% and 97%, respectively. Accumulation over 2.5 days of bid dosing with adavosertib was approximately 2.3 and 2.1 for AUC_0–12_ and C_max_, respectively. The adavosertib concentration in human plasma ranged from 4 to 2000 nM. Precision (%CV) was ≤ 4.2% and accuracy (percentage bias) was within 0.7–1.5% for the QC samples. Reproducibility was confirmed by re-analysis of 87 study samples (with adavosertib concentrations above the LLOQ) selected at random; 98.9% of the results obtained following the initial and repeat analyses were within 20.0% of the mean of the two values, thus meeting the acceptance criteria [23].

**Table 3:** Summary of pharmacokinetic parameters of adavosertib following co-administration of cocktail (pharmacokinetic analysis set)

	AUC_0–12_, nM·h	C_max_, nM	C_min_, nM	C_avg_, nM	t_max_, h	CL_ss_/F, L/h	FI, %
Geometric mean, *n* = 16 (*N* = 22)	12,170	1437	567	1018	ND	ND	ND
CV, %	31.9	29.0	36.7	32.2	ND	ND	ND
Arithmetic mean (SD) (*n* = 16, *N* = 22)	12,720 (3796)	1490 (398)	602 (219)	1065 (319)	ND (ND)	38.0 (12.11)	85.7 (24.3)
Median (minimum, maximum)	11,970 (6950, 18,200)	1415 (853, 2120)	563 (344, 1050)	991 (580, 1540)	3.0 (0.75, 6.00)	36.9 (24.3, 63.5)	79.8 (54.3, 135.8)

Treatment: adavosertib + cocktail: caffeine (200 mg tablet), omeprazole (20 mg capsule), and midazolam (1 mL of 2 mg/mL syrup formulation) on day 3. CV calculated as 100 x √[exp(s^2^)–1], where s is the SD of the data on a log scale. *AUC*_*0–12*_ area under the plasma concentration–time curve from time zero to 12 h, *C*_*avg*_ apparent average plasma concentration over a dosing interval, *CL*_*ss*_*/F* apparent clearance at steady state, *C*_*max*_ maximum plasma drug concentration, *C*_*min*_ minimum plasma drug concentration, *CV* coefficient of variance, *FI* fluctuation index over a dosing interval, *ND* not determined, *SD* standard deviation, *t*_*max*_ time to reach maximum plasma concentration

### Safety

Causally related AEs (stratified by any grade and grade ≥ 3) are shown in Supplementary Table 2 for patients who received only adavosertib and patients who received both adavosertib and the probe cocktail. Four of 33 (12.1%) patients experienced AEs with cocktail alone, compared with 16/26 (61.5%) patients who received probe cocktail plus adavosertib and 16/30 (53.3%) patients after receiving adavosertib alone on days 1 and 2. Treatment-related AEs were reported by 12/26 (46.2%) patients receiving probe cocktail plus adavosertib and 11/30 (36.7%) patients after receiving adavosertib alone on days 1 and 2. The most common treatment-related AEs in patients receiving probe cocktail plus adavosertib (*n* = 26 patients) were diarrhea and nausea (both in four [15.4%] patients) and dizziness (in two [7.7%] patients). The most common treatment-related AEs in patients receiving adavosertib alone (*n* = 30 patients) were diarrhea (in 10 [33.3%] patients), vomiting (in six [20.0%] patients), and nausea (in three [10.0%] patients).

Adverse events of CTCAE grade ≥ 3 were observed in: 4/26 (15.4%) patients receiving probe cocktail plus adavosertib (anemia, neutropenia, pulmonary embolism, and portal vein thrombosis in one [3.8%] patient each); 5/30 (16.7%) patients receiving adavosertib alone on days 1 and 2 (diarrhea in three [10.0%] patients, necrotizing soft-tissue infection, neutropenia, thrombocytopenia, dehydration, hypokalemia, hypoxia, nausea, pancreatitis, small-intestinal obstruction, vomiting, and acute kidney injury in one [3.3%] patient each [multiple events were experienced by the same five (16.7%) patients]); and 0/33 patients receiving cocktail alone.

One patient receiving adavosertib alone on days 1 and 2 reported two serious AEs (SAEs) considered related to adavosertib (acute kidney injury, which resolved after 2 days, and pancreatitis, which did not resolve during the study but improved to grade 2 after 2 days) that led to discontinuation; four patients reported five SAEs that were not considered related to treatment (grade 2 bacterial pneumonia and deep vein thrombosis, which did not resolve; grade 3 necrotizing soft-tissue infection, which resolved; grade 3 small-bowel obstruction, which did not resolve; and grade 4 pulmonary embolism, which resolved).

Two patients discontinued treatment because of AEs: one as a result of grade 3 diarrhea considered related to adavosertib, which resolved, and the other for grade 3 small-bowel obstruction, which was not considered related to adavosertib and did not resolve.

No AEs with an outcome of death were observed; one patient died as a result of disease progression (pancreatic cancer) following receipt of the probe cocktail but prior to receiving adavosertib.

## Discussion

In this study, adavosertib exposure, when given alone or co-administered with the cocktail drugs, was similar to that observed in previous studies when given alone [5]. Therefore, concomitant administration of drugs metabolized by CYP3A, CYP2C19, and CYP1A2 is unlikely to have a significant clinical effect on adavosertib PKs. Adavosertib, when given bid at a dose of 225 mg for 2.5 days, exhibited low intra-patient variability (eg < 10% for AUC_0–12_ and C_max_), and adavosertib accumulation over 2.5 days of bid dosing was approximately twofold. The detailed PK data from this study reveal that the point estimates of the AUC_0–12_ and AUC_0–t_ geometric LS mean ratios of all three probe cocktail drugs in the presence of adavosertib (225 mg bid) compared with probe cocktail drugs administered alone showed a 1.25- to < twofold increase [17]. Adavosertib therefore meets the US Food and Drug Administration (FDA) guidance for definition of a weak inhibitor of CYP1A2, CYP2C19, and CYP3A [17].

A physiologically based PK (PBPK) model (based on in vitro studies of absorption, distribution, metabolism, and excretion, clinical adavosertib PK data for doses of 175–300 mg once daily [qd] or bid, and multiple dosing schedules [3 days on/4 days off and 5 days on/9 days off]) to assess multifaceted CYP modulation predicted that adavosertib is mainly metabolized by CYP3A with an FMO3 and/or FMO5 component and is a time-dependent (irreversible) inhibitor of CYP3A and a weak reversible (direct) inhibitor of CYP2C8, CYP2C9, and CYP2C19 [16, 24]. Model predictions of adavosertib being a weak reversible inhibitor of CYP2C19 were confirmed by this study. Concentration–time curves for midazolam, the substrate of CYP3A, and its metabolite 1ʹ-HM show both midazolam and 1ʹ-HM plasma concentrations increasing then returning towards baseline/elimination over the sampling period. The PBPK model predictions of adavosertib being a weak reversible inhibitor of CYP2C8 and CYP2C9 [16, 24] remain clinically unvalidated but are in line with observed effects of adavosertib being a weak inhibitor of CYP1A2, CYP2C19, and CYP3A.

The observed safety profile of adavosertib in this study was concordant with that found in previous clinical studies [7, 15]. The most prevalent AEs were gastrointestinal toxicities (diarrhea, vomiting, and nausea). No new or unexpected adavosertib-related safety concerns were identified.

Although the study was terminated early because of the difficulty in enrolling patients (i.e. screening/rescreening failures) and evaluable data not being available for all enrolled patients (as a result of protocol deviations/events), this had minimal impact on the interpretation of the PK results. Overall, the data from evaluable patients were adequate and representative of the primary objective of assessing the DDI potential of adavosertib. Instances of pre-dose concentrations > 5% of C_max_ were especially prevalent for caffeine and paraxanthine, despite the study design including washout periods and restriction of caffeine consumption. A potential explanation for this finding is that caffeine/paraxanthine elimination may be slower in this patient population than in participants in previous DDI studies; also, and perhaps the most likely explanation, patients may not have been strictly compliant with the caffeine restrictions in the protocol. The true exposure increase when caffeine/paraxanthine were co-administered with adavosertib may therefore be lower than reported here. Compared with the recommended phase II dose for monotherapy (300 mg qd, 5 days on/2 days off for 2 of 3 weeks) and the maximum tolerated dose of monotherapy (125 mg bid, 5 days on/9 days off for 2 of 3 weeks), both of which were determined after the present study commenced, the adavosertib dose in this study (225 mg bid) was higher and the dosing duration (2.5 days) sufficient to reach steady state; therefore, DDIs at the recommended phase II dose for monotherapy are not expected to be worse than observed during this study [25]. The relatively small study sample size facilitates the balance of needing to gain adequate PK data against exposing as few patients as possible to study procedures.

Overall, the results from this study suggest that adavosertib is a weak inhibitor of CYP1A2, CYP2C19, and CYP3A according to the US FDA guidance for definition of a weak inhibitor [17]. Although the limited impact on probe drug exposures suggests that it is unlikely that dose adjustments of drugs metabolized by CYP1A2, CYP2C19, and CYP3A will be needed when co-administered with adavosertib, adequate safety monitoring and dose modification may be necessary, particularly when co-administration occurs with substrates with a narrow therapeutic index.

## Conclusions

Adavosertib, when administered at a dose of 225 mg bid, is a weak inhibitor of CYP1A2, CYP2C19, and CYP3A and therefore has a low risk of causing clinically significant DDIs with drugs metabolized by these enzymes.

## Supplementary Information

Below is the link to the electronic supplementary material.

Supplementary file1 (PDF 304 kb)

## Data Availability

Data underlying the findings described in this manuscript may be obtained in accordance with AstraZeneca’s data sharing policy described at https://astrazenecagrouptrials.pharmacm.com/ST/Submission/Disclosure. Data for studies directly listed on Vivli can be requested through Vivli at www.vivli.org. Data for studies not listed on Vivli could be requested through Vivli at https://vivli.org/members/enquiries-about-studies-not-listed-on-the-vivli-platform/. AstraZeneca Vivli member page is also available outlining further details: https://vivli.org/ourmember/astrazeneca/.
