# Supplementary material for: Phase I study to assess the effect of adavosertib (AZD1775) on the pharmacokinetics of substrates of CYP1A2, CYP2C19, and CYP3A in patients with advanced solid tumors
Source: Cancer Chemother Pharmacol. 2023 Jul 2;92(3):193–203. doi: 10.1007/s00280-023-04554-3 (PMC10363032; doi:10.1007/s00280-023-04554-3)
Supplement: Supplementary file 1 — Supplementary file1 (PDF 304 kb) [file 280_2023_4554_MOESM1_ESM.pdf]

Supplementary material

Phase I study to assess the effect of adavosertib (AZD1775) on the pharmacokinetics of substrates of CYP1A2, CYP2C19, and CYP3A in patients with advanced solid tumors

Någård M, Ah-See M-L, Strauss J, et al.

Supplementary methods

- Patients meeting the following criteria were excluded:
  - Use of any anticancer treatment drug ≤21 days or ≤5 half-lives (whichever is shorter) prior to the first dose of adavosertib; for drugs for which five half-lives is ≤21 days, a minimum of 10 days between termination of the prior treatment and administration of adavosertib treatment was required
  - No other anticancer therapy (chemotherapy, immunotherapy, hormonal anticancer therapy, radiotherapy [except for palliative local radiotherapy]), biological therapy, or novel agent was permitted while receiving study treatment
  - Herbal preparations taken within 7 days of beginning study treatment (ie first administration of cocktail [day −8]); in the case of St John's wort, patients must not have taken this herbal preparation ≤21 days prior to the first dose of cocktail (day −8); in the case of Angelica root (bai zhi), patients must not have taken this herbal preparation 2 weeks prior to beginning study treatment
  - Any proton pump inhibitor (omeprazole, lansoprazole, esomeprazole, pantoprazole, etc) taken within 7 days of beginning study treatment (ie first administration of cocktail [day −8])
  - Inability to withhold antacids for 6 hours or histamine H_2 antagonists (cimetidine, ranitidine, famotidine, nizatidine) for up to 96 hours at a time (24 hours prior to and following both cocktail administration and adavosertib administration)
  - Any intake of grapefruit, grapefruit juice, Seville oranges, Seville orange marmalade, or other products containing grapefruit or Seville oranges within 7 days of the start of treatment (ie first administration of cocktail [day −8])
  - Excessive intake of caffeine and/or consumption of any caffeine-containing drinks or food within 36 hours of administration of the cocktail on day −8
  - Receipt of prescription or non-prescription drugs or other products:

    - Known to be sensitive to cytochrome P450 (CYP) 3A4 substrates, or CYP3A substrates with a narrow therapeutic index that could not be discontinued 2 weeks prior to the first administration of adavosertib (day 1) and withheld throughout the study until 2 weeks after the last administration of adavosertib
    - Known to be moderate to strong inhibitors/inducers of CYP3A that could not be discontinued 2 weeks prior to beginning study treatment (ie first administration of cocktail [day −8]) and withheld throughout the study until 2 weeks after the last administration of adavosertib
  - Adjustments to prescription or non-prescription drugs or other products known to be mild inhibitors or inducers of CYP3A within 1 week prior to the first dose of the cocktail (day −8)
  - Receipt of prescription or non-prescription drugs or other products known to be moderate to strong inhibitors/inducers of CYP1A2 or CYP2C19 that could not be discontinued 2 weeks prior to beginning study treatment (ie first administration of cocktail [day −8]) and withheld through day 4
  - Receipt of midazolam and/or omeprazole (or esomeprazole) within 14 days of beginning study treatment (ie first administration of cocktail [day −8])
- Co-administration of aprepitant or fosaprepitant during this study was prohibited
- Oral contraceptives were approved with the medical monitor to ensure that there were no interactions between the oral contraceptive and cocktail of drugs prior to trial entry

Supplementary results

Supplementary Fig. S1 Geometric mean (± SD) plasma concentration of (**a**) paraxanthine, (**b**) 5-hydroxyomeprazole, and (**c**) 1'-hydroxymidazolam

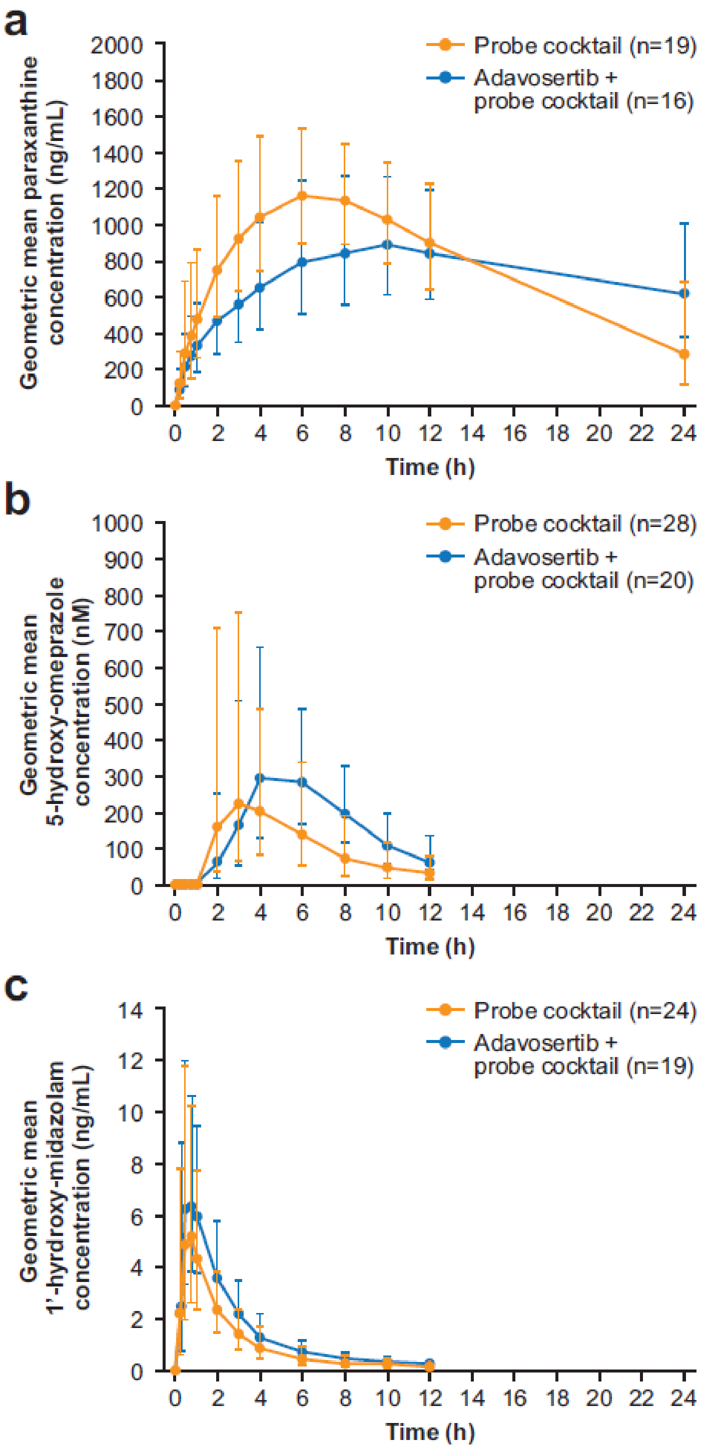

Exponential of (mean of log concentration ± SD of log concentration). Probe cocktail: caffeine (200 mg tablet), omeprazole (20 mg capsule), and midazolam (1 mL of 2 mg/mL syrup formulation) on day −8. Adavosertib + probe cocktail: cocktail and adavosertib 225 mg (3 x 75 mg capsules) on day 3. *SD* standard deviation

Supplementary Table S1. Evaluable patients for substrates and metabolites (PK analysis set, *N* = 33)

Substrate/metabolite (*n* dosed)	Patients with evaluable data, *n* (%)
Caffeine	28 (85)
Period 1 (33)	25 (76)
Period 2 (24)	19 (58)
Periods 1 and 2	16 (48)
Paraxanthine	23 (70)
Period 1 (33)	19 (58)
Period 2 (24)	16 (48)
Periods 1 and 2	12 (36)
Omeprazole	29 (88)
Period 1 (33)	27[a] (82)
Period 2 (24)	20 (61)
Periods 1 and 2	19 (58)
5-HO	29 (88)
Period 1 (33)	28 (85)
Period 2 (24)	20 (61)
Periods 1 and 2	19 (58)
Midazolam	24 (73)
Period 1 (33)	23 (70)
Period 2 (24)	19 (58)
Periods 1 and 2	18 (55)
1'-HM	25 (76)
Period 1 (33)	24 (73)
Period 2 (24)	19 (58)
Periods 1 and 2	18 (55)

[a]28 patients were evaluable, but one patient had an insufficient number of quantifiable concentrations to allow the calculation of PK parameters

1'-HM 1'-hydroxymidazolam, *5-HO* 5-hydroxyomeprazole, *PK* pharmacokinetic

Supplementary Table S2. Causally related AEs (safety analysis set)

Preferred term	Number of patients (%)					
	Adavosertib[a] AEs of any grade (n = 30)	Adavosertib[a] grade ≥3 AEs (n = 30)	Adavosertib + cocktail[b] AEs of any grade (n = 26)	Adavosertib + cocktail[b] grade ≥3 AEs (n = 26)	Total[c] AEs of any grade (n = 30)	Total[c] grade ≥3 AEs (n = 30)
Any AE[d]	11 (36.7)	4 (13.3)	12 (46.2)	2 (7.7)	19 (63.3)	6 (20.0)
Blood and lymphatic system disorders	2 (6.7)	1 (3.3)	2 (7.7)	2 (7.7)	4 (13.3)	3 (10.0)
Anemia	0	0	1 (3.8)	1 (3.8)	1 (3.3)	1 (3.3)
Neutropenia	1 (3.3)	1 (3.3)	1 (3.8)	1 (3.8)	2 (6.7)	2 (6.7)
Thrombocytopenia	1 (3.3)	0	1 (3.8)	0	2 (6.7)	0
Metabolism and nutrition disorders	2 (6.7)	2 (6.7)	1 (3.8)	0	3 (10.0)	2 (6.7)
Decreased appetite	0	0	1 (3.8)	0	1 (3.3)	0
Dehydration	1 (3.3)	1 (3.3)	1 (3.8)	0	2 (6.7)	1 (3.3)
Hypokalemia	1 (3.3)	1 (3.3)	0	0	1 (3.3)	1 (3.3)
Nervous system disorders	0	0	2 (7.7)	0	2 (6.7)	0
Dizziness	0	0	2 (7.7)	0	2 (6.7)	0
Vascular disorders	0	0	1 (3.8)	0	1 (3.3)	0
Flushing	0	0	1 (3.8)	0	1 (3.3)	0
Respiratory, thoracic and mediastinal disorders	0	0	1 (3.8)	0	1 (3.3)	0
Nasal ulcer	0	0	1 (3.8)	0	1 (3.3)	0

Gastrointestinal disorders	11 (36.7)	3 (10.0)	8 (30.8)	0	17 (56.7)	3 (10.0)
Diarrhea	10 (33.3)	3 (10.0)	4 (15.4)	0	14 (46.7)	3 (10.0)
Nausea	3 (10.0)	1 (3.3)	4 (15.4)	0	6 (20.0)	1 (3.3)
Pancreatitis	1 (3.3)	1 (3.3)	0	0	1 (3.3)	1 (3.3)
Stomatitis	0	0	1 (3.8)	0	1 (3.3)	0
Vomiting	6 (20.0)	1 (3.3)	1 (3.8)	0	7 (23.3)	1 (3.3)
Musculoskeletal and connective tissue disorders	0	0	1 (3.8)	0	1 (3.3)	0
Muscle spasms	0	0	1 (3.8)	0	1 (3.3)	0
Renal and urinary disorders	1 (3.3)	1 (3.3)	0	0	1 (3.3)	1 (3.3)
Acute kidney injury	1 (3.3)	1 (3.3)	0	0	1 (3.3)	1 (3.3)
General disorders and administration site conditions	0	0	1 (3.8)	0	1 (3.3)	0
Fatigue	0	0	1 (3.8)	0	1 (3.3)	0
Investigations	2 (6.7)	0	2 (7.7)	0	4 (13.3)	0
Blood creatinine increased	0	0	1 (3.8)	0	1 (3.3)	0
Weight decreased	2 (6.7)	0	1 (3.8)	0	3 (10.0)	0

Cocktail: caffeine (200 mg tablet), omeprazole (20 mg capsule), and midazolam (1 mL of 2 mg/mL syrup formulation). No patients who received cocktail alone experienced causally related AEs (AE counted if onset was after first dose of cocktail drug and prior to first dose of adavosertib and up to and including 30 days after last dose if patient discontinued before first dose of adavosertib)

[a]Patients who received adavosertib; AE counted if onset was after first dose of adavosertib and prior to second dose of cocktail drug or up to and including 30 days after last dose whereby patient did not receive second dose of cocktail drug

[b]Patients who received adavosertib and cocktail; AE counted if onset was after second dose of cocktail drug, or up to and including 30 days post the last administration where the patient did not enter part B

[c]Patients who received adavosertib 225 mg (3 x 75 mg capsules) twice daily on days 1 and 2, and cocktail on day 3, and adavosertib 225 mg (3 x 75 mg capsules) on day 3

[d]Some patients experienced multiple AEs (as listed)

AE adverse event
